# The Effect of Glycerin Content in Sodium Alginate/Poly(vinyl alcohol)-Based Hydrogels for Wound Dressing Application

**DOI:** 10.3390/ijms222112022

**Published:** 2021-11-06

**Authors:** Katarzyna Bialik-Wąs, Klaudia Pluta, Dagmara Malina, Mateusz Barczewski, Katarzyna Malarz, Anna Mrozek-Wilczkiewicz

**Affiliations:** 1 Department of Organic Chemistry and Technology, Faculty of Chemical Engineering and Technology, Cracow University of Technology, 24 Warszawska St., 31155 Cracow, Poland; katarzyna.bialik-was@pk.edu.pl; 2 Department of Chemical Technology and Environmental Anatylics, Faculty of Chemical Engineering and Technology, Cracow University of Technology, 24 Warszawska St., 31155 Cracow, Poland; dagmara.malina@pk.edu.pl; 3 Faculty of Mechanical Engineering and Management, Institute of Materials Technology, Poznan University of Technology, 24 Jana Pawła II St., 60965 Poznan, Poland; mateusz.barczewski@put.poznan.pl; 4 A. Chelkowski Institute of Physics and Silesian Center for Education and Interdisciplinary Research, University of Silesia in Katowice, 75 Pułku Piechoty 1, 41500 Chorzow, Poland; katarzyna.malarz@us.edu.pl (K.M.); anna.mrozek-wilczkiewicz@us.edu.pl (A.M.-W.)

**Keywords:** wound dressings, sodium alginate/poly(vinyl alcohol) matrices, hydrogels, glycerin

## Abstract

The impact of different amounts of glycerin, which was used in the system of sodium alginate/poly(vinyl alcohol) (SA/PVA) hydrogel materials on the properties, such as gel fraction, swelling ability, degradation in simulated body fluids, morphological analysis, and elongation tests were presented. The study shows a significant decrease in the gel fraction from 80.5 ± 2.1% to 45.0 ± 1.2% with the increase of glycerin content. The T_5_ values of the tested hydrogels were varied and range from 88.7 °C to 161.5 °C. The presence of glycerin in the matrices significantly decreased the thermal resistance, which was especially visible by T_10_ changes (273.9 to 163.5 °C). The degradation tests indicate that most of the tested materials do not degrade throughout the incubation period and maintain a constant ion level after 7-day incubation. The swelling abilities in distilled water and phosphate buffer solution are approximately 200–300%. However, we noticed that these values decrease with the increase in glycerin content. All tested matrices are characterized by the maximum elongation rate at break in a range of 37.6–69.5%. The FT-IR analysis exhibits glycerin changes in hydrogel structures, which is associated with the cross-linking reaction. Additionally, cytotoxicity results indicate good adhesion properties and no toxicity towards normal human dermal fibroblasts.

## 1. Introduction

Every year, innovative technologies and possibilities in the area of medical and pharmaceutical research make it possible to develop new approaches in the treatment of slow-healing wounds and other dermatological disorders. Generally, infections in the healing wound are very common, especially in the case of patients with diabetes, and they are characterized by a variety of treatment methods. Very often, such wounds can become chronic, which can lead to a need for long-term treatment [1,2,3,4]. In this situation, traditional methods of rehabilitation may be insufficient, and that is why a modern product such as an interactive wound dressing should be used [4,5]. Different types of dressing materials are commercially available, such as: Algisite M, Tegaderm™ hydrocolloid dressing, Evicel^®^, Coseal^®^, and Elasto-Gel™ [3,6]. Nowadays, there is a high availability of wound treatment options that contain additional natural components, such as: manuka honey [7,8,9], *Aloe vera* [10,11,12,13], *Echinacea purpurea* [14], *Calendula officinalis* [11], *Centella* *asiatica* [15], *Azadirachta indica* [16,17], *Tecomella undulate* [18,19], *Hypericum perforatum* [20,21], *Garcinia mangostana* [22,23], *Tectona grandis* [24] as well as glycerin [25,26,27]. The active substances (saponins, tannins, flavonoids, alkaloids and quinones) that are present in the herbal extracts or hydrolats can ensure further therapeutic effects, such as antimicrobial, antioxidant, antibacterial and anti-inflammatory [28,29,30].

In the case of Elasto-Gel™ dressings, the most important component is glycerin combined with a hydrophilic polymer. This type of product can be used in the case of some 15–20-year-old chronic wounds, but it is not suitable for third-degree burns, which results from its antimicrobial activities. It has been proven that the efficiency of 85% glycerin solutions is characterized by slow bactericidal and very high virucidal activity [6]. Glycerin (the simplest trihydric alcohol, also called glycerol) is a well-known natural humectant that can bind and hold moisture at the place of application. Additionally, it can play a role of denaturant, a fragrance ingredient, a hair conditioning agent, an oral health-care drug, a skin protectant and conditioning agent as well as a viscosity-decreasing substance [31,32]. It can be produced from natural sources, such as animals and plants, and synthetically from nontriglyceride. The latest research of the Cosmetic Ingredient Review Expert Panel confirmed that glycerin is a safe component of products [31,33]. Due to this categorization glycerin is commonly used in various dermatological and cosmetic products. It turns out that it was used in 15,654 cosmetic products, including 862 materials for application around the eye, 160 lipsticks, 369 hair dyes and colours, 1259 bath soaps and detergents, 7756 skincare products, and 244 suntan preparations [31,34].

Interestingly, glycerin present in the three-dimensional network of a polymeric matrix can absorb excess exudate and prevent its pooling in the wound or on the surrounding skin, ensuring a proper wound healing environment [6]. Hence, many researchers incorporated glycerin into hydrogels, which improved their toughness, transparency, conductivity and thermoplasticity [35,36,37,38,39]. Various possible combinations of glycerin with the crosslinked polymers, such as: methoxyl pectin/gelatin/carboxymethyl cellulose [40], chitosan/hydroxypropyl methylcellulose [41], polyacrylamide/gelatin/ε-polylysine [42], and PVA/sodium alginate [43] have been observed.

Therefore, we focused on the determination of the effect of different glycerin concentrations on the physicochemical, structural, morphological, thermal, mechanical and biological properties of sodium alginate/poly(vinyl alcohol) hydrogel materials.

## 2. Results and Discussion

The transparency of a hydrogel dressing gives the opportunity to monitor the progress of the wound healing process without the removal of the dressing. The study shows that with the addition of glycerin into the SA/PVA system, the hydrogel became transparent. Moreover, it could be seen that the sample containing 1.7% (*v/v*) of glycerin (S2G0.5) still retained high transparency, while the transparency of S2G0 without glycerin drastically decreased. The same observation was previously reported by Hu et al., who prepared a poly(vinyl alcohol)/sodium alginate/glycerol organohydrogel electrolyte [35]. Additionally, due to the addition of glycerin to the system, the flexibility of the samples changed. Without glycerin, the S2G0 hydrogel became more compact and stiffer.

### 2.1. Gel Fraction

Crosslinking is one of the important processes determining the properties of obtained hydrogel materials. The gel fraction value gives information about the effectiveness of the crosslinking process, forming the insoluble fraction. This parameter affects the integrity of the polymeric network structure and thus prescribes the mechanical and swelling properties. The gel fractions were calculated, and the results are presented in Table 1.

The study showed that increasing PVA concentration noticeably increased the gel fraction, and at the same time it can be seen that an increase in SA concentration slightly strengthens the gelation process of the materials. Increasing values of gel fraction imply that this parameter is mostly dependent on the applied concentration of both ingredients and that the increase of SA and PVA content leads to better crosslinking. In addition, the gel fraction is definitely higher for glycerin-free hydrogel (S2G0), which reached the value of about 80% as compared to S2G1 with the same SA and PVA concentration, indicating a clear effect on the gelation process. This effect can also be observed when glycerin is used at a concentration of 1.7% (*v/v*). This may suggest that glycerin addition reduces the efficiency of the hydrogel cross-linking process and thus affects the integrity of the polymeric network. Similar observations were reported by Gwon et al., who described PVA/glycerin hydrogel preparation with the use of γ-irradiation [44].

### 2.2. Determination of Swelling Behaviour

**Figure 1:**
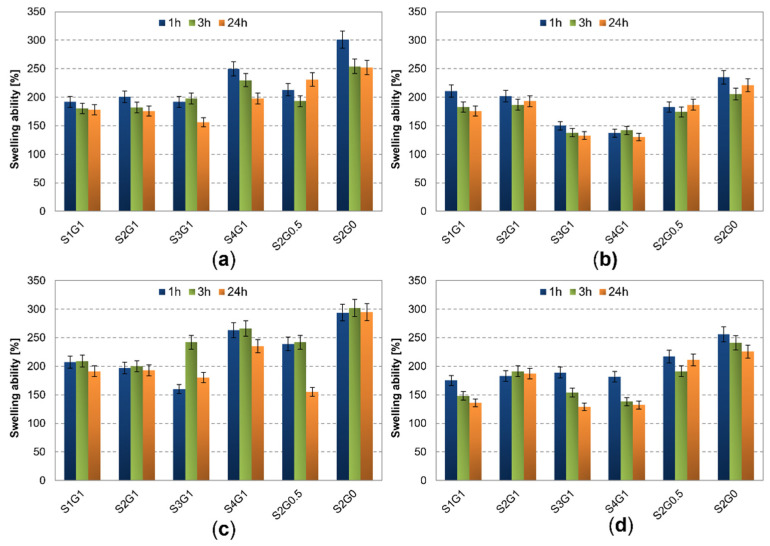
Effect of hydrogel composition on swelling degree after immersing in (**a**) distilled water at ambient temperature; (**b**) distilled water at 37 °C; (**c**) PBS at ambient temperature; (**d**) PBS at 37 °C (*n* = 3).

The ability of a hydrogel to preserve fluids is an important aspect when evaluating its value for dressing materials. Fluid absorption capacity depends on critical factors such as hydrogel composition, and the external stimulus which includes pH, temperature, and the type of fluids, as shown in Figure 1.

The behavior of the materials in distilled water or phosphate-buffered saline (PBS) indicates a well-chosen composition of the base matrix, as hydrogel swelling values were obtained in accordance with expectations and previous studies by the authors on the preparation of SA/PVA-based hydrogels [12,14,45]. None of the tested hydrogels show swelling capacity above 300%; moreover, in each case studied, after an initially rapid water uptake, the hydrogels equilibrated the fluid absorption, reaching an equilibrium state by the end of the experiment. Nevertheless, some dependence on hydrogel composition, immersion temperature or fluid type was observed. Considering the materials differing in SA and PVA concentrations, no significant effect of the concentration of the solutions used was observed during the preparation of the mixtures—in each variant the behavior of the materials was similar. However, it was observed that the presence of glycerin significantly altered the tendency of the materials to swell, leading to rapid fluid uptake by the materials without polyol addition of up to about 100% more than for the materials containing glycerin—a trend of decreasing swelling with the addition of glycerin is clearly noticeable. The dry hydrogel, without glycerin, is hard and inflexible and takes up fluid more readily, in contrast to the glycerin-containing materials, which are soft and flexible. This is likely due to the fact that glycerin increases the moisture content of the materials by binding water, which provides flexibility and also limits fluid absorption in excess. In addition, the lower swelling capacity values may also be observed due to the fact that the addition of acidic glycerin with a pH value of 5 [46] reduces the affinity of alginate carboxylate anions for water, which in turn reduces the propensity for fluid uptake [14]. The glycerin trend was observed regardless of incubation temperature or fluid used. The above observations allow us to draw a conclusion that in the case of designing dressing materials that are to absorb a large exudate from the wound, hydrogels with a lower content of glycerin could work efficiently. Nevertheless, if the wound does not leak too intensively, the use of higher concentrations of glycerin would result in a prolonged therapeutic effect by ensuring the optimal level of moisturization of the wound surrounding. It is also worth noting that the type of fluid and immersion temperature slightly affect the swelling value of tested hydrogels. Hydrogels swell less in PBS fluid than in water, while lower values are obtained during immersion at an elevated temperature for each type of material, regardless of the composition.

### 2.3. Degradation Tests

For hydrogels with potential biomedical applications, their degradation when in contact with body fluids is a key functional parameter. During the release of transdermal therapeutic substances, it is important to ensure a controlled release of active substances, with a gradual degradation rate.

**Figure 2:**
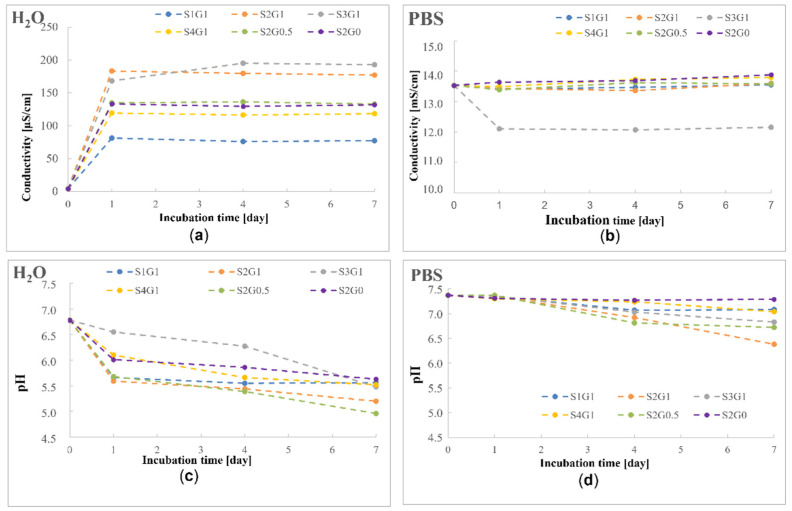
The changes in conductivity and pH value during the 7-day immersion at 37 °C. (**a**) Conductivity changes in distilled water; (**b**) conductivity changes in PBS; (**c**) pH changes in distilled water; (**d**) pH changes in PBS.

A 7-day incubation of the hydrogels in distilled water demonstrated the stability over time of all materials analyzed (Figure 2), regardless of matrix composition and glycerin content, which also confirms the authors’ previous observations regarding the tendency of SA/PVA hydrogels to degrade [12,14,45]. After 24 h of the experiment, the conductivity of distilled water increased significantly (from 4.2 µS/cm), reaching the lowest value for sample S1G1 (~80 µS/cm) and a value 2.5 times higher for materials S2G1 and S3G1 (~160–180 µS/cm). These changes were due to the fact that the ion-free medium tends to reach an equilibrium state upon contact with the material, which is maintained from the time it reaches 24 h of immersion until the end of the experiment, meaning that the materials do not rapidly change or decompose over time. An interesting relationship was observed for materials incubated in PBS fluid containing numerous ions, simulating the internal environment of an organism. All materials except hydrogel containing 10% PVA, 1.5% SA, and 3.4% (*v/v*) glycerin (S3G1) did not degrade throughout the incubation period and maintained a constant ion level, close to the initial value (13.53 mS/cm). On the contrary, the presence of the mentioned material after 24 h led to a decrease in PBS fluid conductivity by 1.5 mS/cm, maintaining a similar value for 7 days of incubation, which may be due to the absorption of ions from the fluid to the hydrogel surface. However, the stability of this parameter over time does not preclude the intended applications. The pH analysis of water and PBS fluid over time supports to draw a general conclusion that all analyzed materials enriched with glycerin lead to a gradual decrease in pH over time, while the absence of glycerol does not significantly affect the pH during the analyzed period. Acidic glycerin is gradually released from the hydrogel into the fluid, lowering the pH from an initial 6.78 for water and 7.37 for PBS fluid by a maximum of about 2 or 1 unit, respectively. The body-like environmental pH, and thus the ions present in the phosphate-buffered saline, have a buffering effect, maintaining the pH at a nearly constant level. It is well known that at a slightly alkaline pH, –COO− groups derived from SA tend to ionize, increasing the amount of fluid absorbed [14]. Gradual lowering of the pH of the environment due to the presence of glycerol inhibits this process so that the materials do not absorb fluid in excess and the swelling values achieved are at the desired level, which was also demonstrated during the analysis of the effect of the presence of glycerol on the swelling capacity of materials in the previous section.

### 2.4. FT-IR Analysis

**Figure 3:**
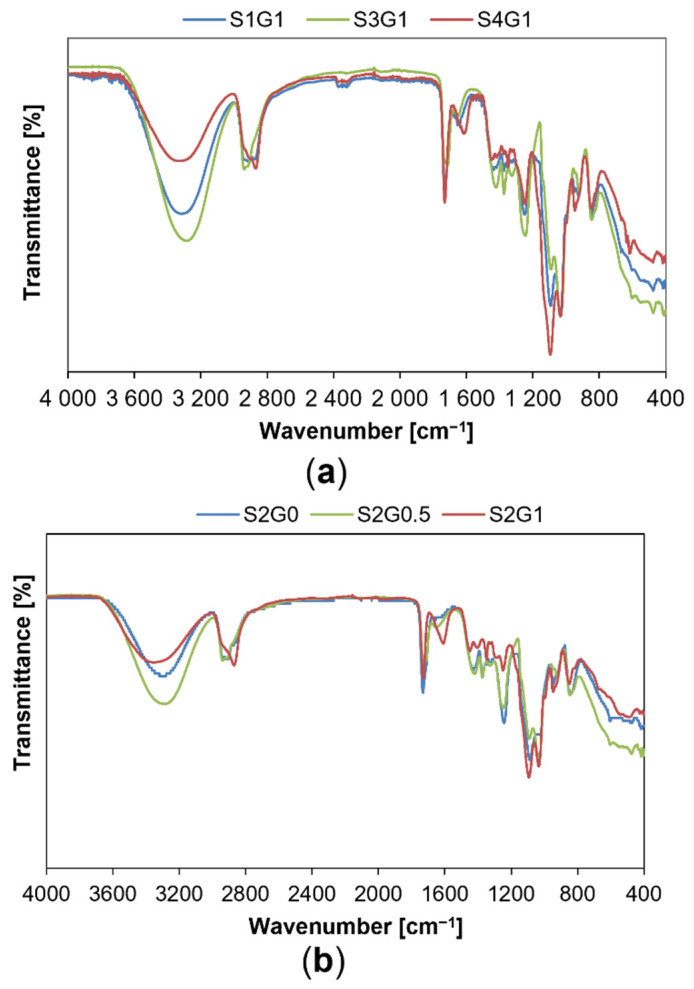
FTIR spectra of hydrogel samples (**a**) with a constant glycerin content (3.4%); (**b**) with varying glycerin content.

Figure 3 compiles the FT-IR spectra of the obtained hydrogels, with Figure 3a showing a summary of the spectra of materials obtained using varying contents of the base matrix components (SA and PVA) with a constant glycerin content (3.4% (*v/v*), while Figure 3b collates the spectra of materials with a constant proportion of alginate and poly(vinyl alcohol), but with varying glycerin content (or none, as a control) in the hydrogel matrix.

Each spectrum in the range 3400–3200 cm^−1^ has the broadest band, which originates from the stretching vibrations of –OH– bonds occurring in all base matrix components—poly(vinyl alcohol), sodium alginate, and glycerin structures [12,14,47,48]. Interestingly, a lower peak intensity was observed for the lower 1.5% alginate content. The next visible peak toward short wavelengths, located at 2930–2820 cm^−1^, is characteristic of the stretching vibrations of C–H from PVA or glycerin. However, the PVA-derived peak is localized at about 2880 cm^−1^ and is more distinct in materials with lower alginate concentrations, while the C–H group vibrations from glycerin are found at about 2680 cm^−1^ [12,48,49]. The subsequently observed peaks are located at lower wavenumbers. The deformation vibrations in the range of 1490–1450 cm^−1^ are characteristic for C–H bonds derived from the –CH_2_– group occurring in the aliphatic PVA chain, and vibrations of O–H bonds at around 1200–1450 cm^−1^ are characterized for the hydroxyl groups of PVA and SA, which interact with each other through hydrogen bonds [45,47,50]. The tensile vibrations from the C–O bond are visible and give peaks in the range 1780–1650 cm^−1^ attributed to carboxyl groups attached to the rings of alginate acids, which can form ester bonds during chemical crosslinking reactions of the hydrogel using PEGDA. Moreover, in the case of the hydrogels obtained by chemical crosslinking using PEGDA, stretching vibrations of the –CH_3_ groups at 2920 cm^−1^, vibrations of C–H and O–H bonds at 1360–1340 cm^−1^, and strong peaks seen in the range 1160–1040 cm^−1^ for the C–O–C are also observed [12,14,47]. The peaks of SA included strong absorption bands at ~1600 cm^−1^ and 1415 cm^−1^, which is related to the asymmetric and symmetrical stretching vibrations of carboxylate anions (–COO−), and the intensity increases with the content of SA in the matrix due to the higher number of free carboxylate anions. Additionally, the bands which appear at 1250 cm^−1^ and 1035 cm^−1^ are attributed to C–O–C in glycosidic bonds of SA. Moreover, bands located at 990 cm^−1^ and 820 cm^−1^ are also observed and they are assigned to the COH bending and –CH_2_ twisting [12,14,45,50,51].

Analysis of Figure 3b with a comparison of spectra depending on the content of glycerin supports a conclusion that the presence of this simple polyol significantly influences the degree of the components’ reactivity and the polymerization process. Glycerin peaks formed at 3600–3000 cm^−1^, ~2990 cm^−1^, 1635 cm^−1^, 1390 cm^−1^, and 1030 cm^−1^ wavelengths, and these peaks belong to –O–H, –C–H, –C–C, C–H, and C–O bonds, respectively [48,49,52].

Generally, the presence of glycerin does not significantly influence the location of peaks, which could suggest a lack of significant changes in the hydrogel structure. However, it turns out that 3.4% (*v/v*) content of glycerin significantly changes the physicochemical properties of the materials. At 1.7% (*v/v*) content, no significant changes were observed and at the same time, the transparency of the product was preserved, which is not the case for the absence of glycerin. The FT-IR spectrum of hydrogel with maximal polyol content shows the presence of a clear peak at ~1630 cm^−1^, originating from the C–C groups present in glycerin, which is likely to significantly influence the degree of substance reactivity during the crosslinking process [48,52]. This was confirmed in the gel fraction study and during swelling studies of the materials. A low value of %*GF* confirms a lower degree of conversion in samples containing glycerin. The change in structure and alterations in the crosslinking process are also confirmed by the peak intensity at ~1240 cm^−1^, corresponding to intermolecular hydrogen interactions between PVA-alginate chains [14,45,50]. A decreasing intensity of the peak with increasing addition of glycerin seems to confirm the above supposition. On the other hand, it is worth mentioning that the highest content of glycerin positively influences the degree of material swelling—the materials enriched with polyol swell less abruptly, and are more elastic and flexible, which can also be caused by changes in hydrogel structure during incomplete crosslinking.

### 2.5. SEM Analysis

**Figure 4:**
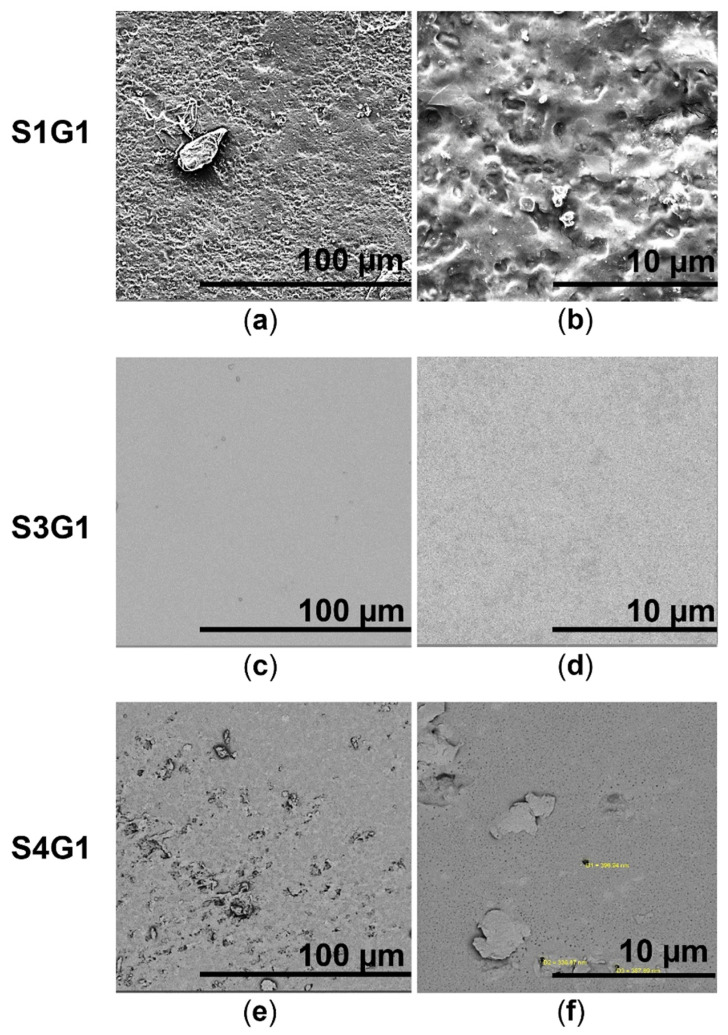
SEM images of hydrogels containing a constant amount of glycerin, such as 3.4% (*v/v*); (**a**,**b**) sample S1G1; (**c**,**d**) sample S3G1; (**e**,**f**) sample S4G1.

**Figure 5:**
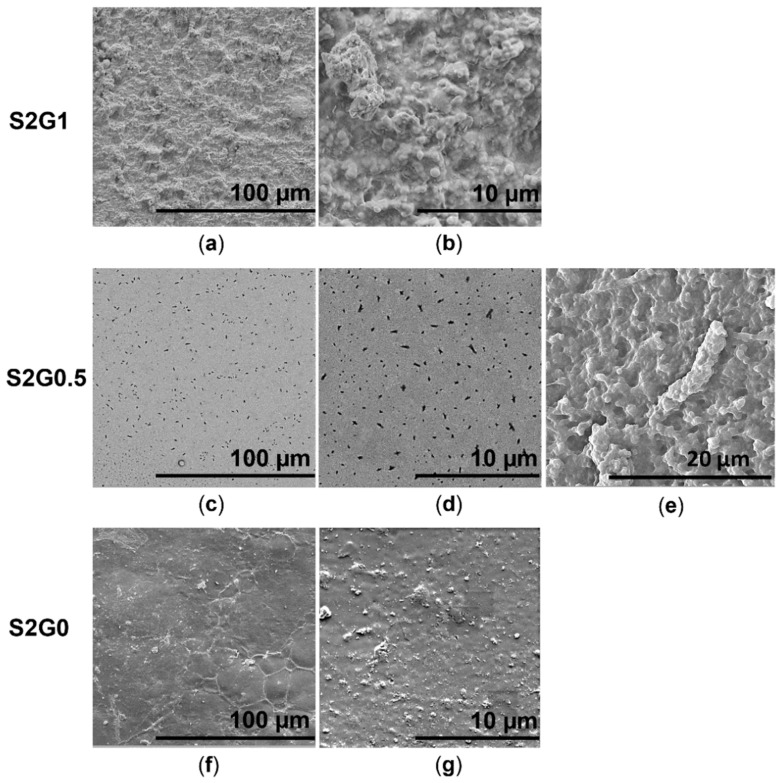
SEM images of hydrogels containing a different glycerin content: 0; 1.7 and 3.4% (*v/v*); (**a**,**b**) sample S2G1; (**c**,**d**) sample S2G0.5; (**e**) cross-section of sample S2G0.5; (**f**,**g**) sample S2G0.

The microstructural properties of the hydrogel wound dressing affect the swelling ability and gaseous exchange, and thus the wound healing process significantly. Accordingly, Figure 4 presents SEM images of hydrogel containing a constant amount of glycerin, such as 3.4% (*v/v*), and Figure 5 shows the results for samples with varying glycerin content: 0; 1.7 and 3.4% (*v/v*).

Despite the use of the same amount of glycerin, which can be seen in Figure 5, the surface of the obtained hydrogels is quite varied. It was caused by the different compositions, especially the concentration of PVA and sodium alginate solutions. When the highest contents of PVA (10%, *w/v*) and the lowest of sodium alginate (1.5%, *w/v*) were used, the surface is the densest and is completely homogeneous without any roughness. However, the structure of sample S3G1 is non-porous. It was observed that the use of 5% (*w/v*) PVA solution (S1G1) changes the surface of analyzed samples to be more irregular when compared to S3G1. However, the most suitable results were obtained for hydrogel that consists of 2% (*w/v*) sodium alginate solution (S4G1), because it is the most porous, which is a positive aspect. Very similar results can be found in the literature [7,12]. Hence, the 5% (*w/v*) PVA solution and 2% sodium alginate were selected for further research with different amounts of glycerin. It was observed that the increase of glycerin content in the system (S2G1) caused more irregularities and some ripples and bumps appeared on the surface. However, we must consider the gel fraction, where we noticed a significant decrease in %*GF* from about 80% to 50% when comparing the sample with the largest amount of glycerin and without additives. Taking this into account, the hydrogel sample S2G0.5 seems the most interesting, because the surface is porous, and the gel fraction is about 63.4 ± 1.8%. Furthermore, SEM analysis of the cross-section of the basic matrix proves that the structure inside the hydrogel is more porous, irregular, and varied; whereas the average pore size is estimated below 5 µm. Generally, the most important aspect is the preparation method, especially the type of crosslinking agent. When chemical crosslinking was used, the hydrogels were characterized by a denser structure than in the case of the ionic methods. Then, the samples exhibited significant porosity, which was confirmed in our previous research and in other literature data [35,45,53].

### 2.6. Thermal Analysis

**Figure 6:**
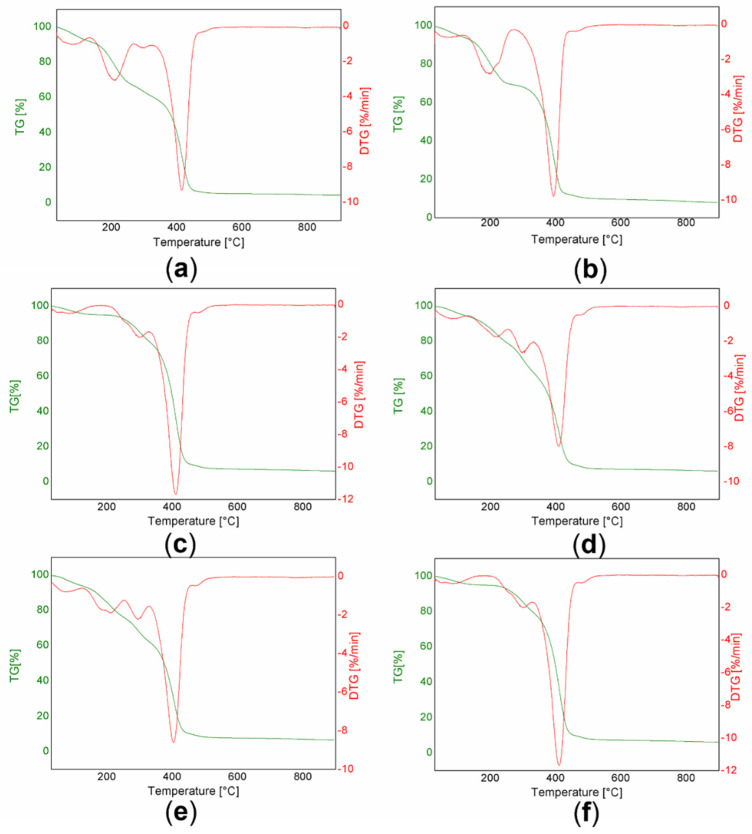
TG and DTG curves of hydrogels; sample (**a**) S1G1; (**b**) S2G1; (**c**) S3G1; (**d**) S4G1); (**e**) S2G0.5 and (**f**) S2G0.

The thermogravimetric analysis (TGA) curves showing the mass loss and rate of mass loss profiles of all samples are presented in Figure 6, while Table 2 compares the characteristic thermal parameters determined from the TG curves for each step in the decomposition sequence of tested hydrogels. In the case of biomedical products, thermal analysis is necessary because it makes it possible to select the appropriate sterilization method.

From the TG and DTG curves, it is observed that the thermal decomposition process took place through 4 consecutive steps. The initial stage of decomposition at around 70–100 °C starts with dehydration of residual water molecules trapped in the hydrogel structure, which is in line with the report by Avella et al. [54]. The second stage observed around 200 °C corresponds to simultaneously occurring effects of the glycosidic bonds cleavage and loss of the adjacent hydroxyl group as water molecules [55]. The TG and DTG curves revealed a high weight loss by a gradual decomposition at the third-step observed at about 300 °C (with small and broad DTG curves) and consecutively the weight loss with a very large and sharp DTG for the final stage (~400 °C), during which the decomposition of the PEGDA network occurs [56]. Interestingly, in the case of the S2G1 sample, no decomposition occurred at a temperature of approximately 300 °C.

The T_5_ values of the tested hydrogel networks ranged from 88.7–161.5 °C and showed a correlation to the concentration of SA and PVA solution as well as to glycerin content. As can be seen with the increasing concentration of the applied PVA and SA solutions, the temperature at which 5% weight loss of each sample occurred increased from 88.7 °C to 117.6 °C. Moreover, the presence of glycerin in the polymeric matrices significantly decreased the thermal resistance, which is especially clear for samples S2G1 and S2G0, where T_10_ is 163.5 °C and 273.9 °C, respectively.

The performed DSC analysis made it possible to conclude that all prepared compositions were characterized by the correct realization of the crosslinking process. No distinct exothermic effects were found for any of the compositions in the temperature range below the degradation temperature described by TGA. The DSC curves representing the heating of the tested materials in the range from −30 to 300 °C are summarized in the Supplementary Information—Figure S1.

### 2.7. Static Tensile Test

The fundamental limitation of the use of hydrogels is their poor mechanical properties. Importantly, ideal hydrogel materials for wound dressing need to satisfy the basic requirements of mechanical stability, which plays a crucial role in creating an optimal environment providing protection from infection.

**Figure 7:**
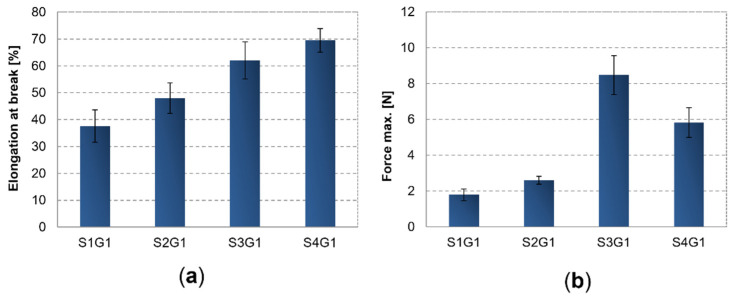
Elongation at break (**a**) and maximum force (**b**) in static stretching test of hydrogels (*n* = 5).

Stretching tests clearly showed the impact of the hydrogels’ compositions on their mechanical properties. All of the tested membranes were characterized by a medium maximum deformation rate of around 37.6–69.5% at break. Importantly, with a constant SA-to-PVA volume ratio, elongation at break increases monotonically with an increasing concentration of both components. As shown in Figure 7, higher elongation at break values were observed in the S4G1 and S3G1 systems, which was closely related to a higher content of PVA leading to an increase in the strength of the polymeric network. It is noteworthy that when the 10% of PVA solution was introduced into the hydrogel system, the elasticity was approximately 24% higher than for samples with 5% of PVA. Moreover, these results are consistent with the measurements of the gel fraction, where the elasticity of the samples increased with an increasing share of the insoluble fraction of hydrogels. This can be explained as being because an effective crosslinking process, expressed by the GF value, leads to the formation of a mechanically stable polymer network with a higher deformation at break value. Furthermore, the stretching test revealed that samples S3G1 and S4G1 containing a greater concentration of PVA showed higher tensile strength, where the highest stress at a break value of around 8.3 N was noted for a matrix with 1.5% of sodium alginate and 10% of poly(vinyl alcohol). Thus, these results suggest that a lower crosslink density of obtained hydrogel systems weakened the tensile strength properties.

### 2.8. Biological Studies

Cytotoxicity is a very important indicator for biological evaluation that is used for biomaterials including hydrogel dressing materials. For this purpose, the MTS assay is used, which is an effective method for assessing cell viability based on the conversion of the MTS tetrazolium compound by the metabolic activity of the viable cells.

**Figure 8:**
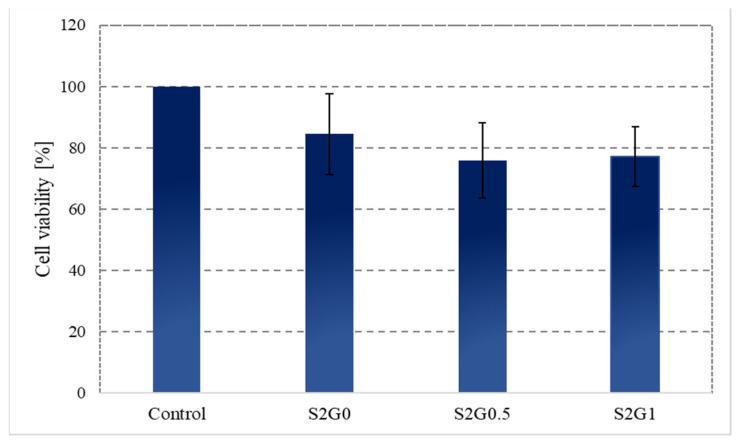
Fibroblasts cell viability seeded onto hydrogel discs (*n* = 3).

Cytotoxicity analysis performed on an in vitro model indicates that the tested samples did not show a toxicity effect towards normal human dermal fibroblasts. Some small differences were observed in the proliferating fraction of cells for sample S2G0 without glycerin and with 1.7% and 3.4% of glycerin. Namely, the addition of glycerin had a negative impact on the proliferation. However, this dependence was barely noticeable. In general, none of the tested samples inhibited the proliferation of the tested cell line significantly, as evidenced by the plot (Figure 8) in which the surviving fraction exceeds 75%. Additionally, it is worth adding that we compared the cells seeded on the culture dish (control) to cells seeded on tested samples. Culture dishes are purposed for cell culturing, and it is clear that the conditions would be better than on tested materials. The positive effects of the PVA/chitosan with glycerin on the growth of L929 cells are also reported by Yang et al. [57]. They found that the morphologies of cells treated with prepared hydrogels were unchanged as compared with the negative samples. Moreover, they demonstrated increased cell proliferation after 48 h incubation.

The used CellTracker™ dye passes through cell membranes and is then converted by esterases present in the cytoplasm of living cells into a fluorescent green cell-impermeant product. This method provides information about cell viability, cell shape, and membrane integrity.

**Figure 9:**
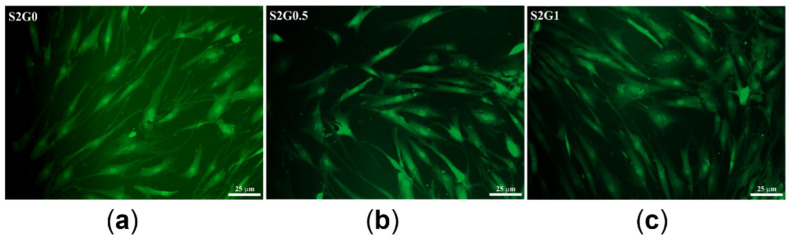
Fibroblast cells stained with CellTracker Green attached to the surface of the hydrogel discs; (**a**) sample S2G0; (**b**) sample S2G0.5; (**c**) sample S2G1. Scale bars indicate 25 µm.

The images obtained from the fluorescence microscope proved that tested samples possessed good adhesion properties, displayed an elongated spindle-shaped morphology, and did not exhibit toxic features. After 72 h of the incubation of NHDF cells on the hydrogel surface, staining with a green fluorescent dye was performed. As shown in Figure 9, no morphological alterations were observed for the cells exposed to tested hydrogels. The cells did not change their shape to round features that might indicate the early process of apoptosis. Moreover, observed cells showed the presence of multiple long cellular protrusions whose length exceeded the size of the cell. The cytoskeleton of observed cells was coherent.

## 3. Materials and Methods

### 3.1. Materials

All chemicals and other substrates used in this study are listed in Table 3 with the name of the producing company, purity degree, and molecular weight.

### 3.2. Fabrication of Hydrogel Materials

The fabrication method of proposed hydrogel materials is based on conventional chemical cross-linking using a 1% solution of ammonium persulfate as an initiator and poly(ethylene glycol) diacrylate (PEGDA, Mn = 700 g/mol) as a cross-linking agent. In order to synthesize the polymer matrices, it is necessary to prepare aqueous solutions of 10 and 5% (*w/v*) of poly(vinyl alcohol) as well as 1.5 and 2% (*w/v*) of sodium alginate. Afterwards, proper amounts of these solutions and a constant amount of poly(ethylene glycol) diacrylate (7.5% *v/v*) were mixed. To investigate the effect of the glycerin addition on the chemical structure and properties of SA/PVA films, a series of hydrogels with 0, 1.7, and 3.4% (*v/v*) of glycerin content were prepared. A detailed description of the hydrogel compositions is shown in Table 4. After that, the prepared mixtures were heated to 70 °C and 4.4% (*v/v*) of ammonium persulfate was added. Next, all specimens were poured into Petri dishes and placed on a heating plate with a temperature of 80 °C for 1.5 h. In this way, a series of polymer films all were prepared. Finally, the materials were conditioned for 24 h in ambient conditions [58].

### 3.3. Gel Fraction

The gel fraction of all hydrogels was measured using samples with dimensions of 10 mm × 10 mm. The samples were initially dried at 40 °C for 24 h and weighed (*W*_0_). The samples were allowed to swell in 30 mL of distilled water for 48 h at ambient temperature until equilibrium swelling was achieved to remove the leachable or soluble parts from the hydrogels. Once equilibrium swelling was attained, samples were again dried at 40 °C for 24 h and weighed (*W_e_*). The gel fraction (%*GF*) was calculated using Equation (1):

(1) $$\%GF=\frac{W_{e}}{W_{0}}\times 100\%,$$

### 3.4. Determination of Swelling Behaviour

The swelling ability is defined as the fractional increase in the weight of the hydrogel materials due to water absorption. Swelling experiments were performed in a phosphate buffer solution (PBS, pH 7.4) and distilled water at ambient temperature and at 37 °C. The crosslinked matrices were cut into 10 mm × 10 mm pieces and subsequently dried and weighed (*W_d_*). Next, each sample was immersed in PBS or distilled water. At specific time intervals, the swollen hydrogels (*W_s_*) were taken out and immediately reweighed after carefully wiping off excess liquid with filter paper. The percentage swelling of the samples was calculated using Equation (2):

(2) $$\%SR=\frac{W_{s}-W_{d}}{W_{d}}\times 100\%,$$

### 3.5. Degradation Tests

The degradation of SA/PVA/glycerin hydrogels was examined in vitro in PBS (initial pH—7.37, conductivity—13.53 mS/cm) as well as in distilled water (initial pH—6.78, conductivity—4.2 µS/cm). In order to prepare materials for degradation testing, the samples were cut into half-gram pieces (in triplicate). Each hydrogel specimen was immersed in 50 mL of immersion solution and then incubated at 37 °C. At specific time intervals, the pH and conductivity values were monitored for each fluid three times during the week. The 1-week incubation time assumes that the resulting dressings would be in contact with the patient’s body for a maximum of 7 days.

### 3.6. FT-IR Analysis

To investigate the chemical structure of the obtained hydrogel materials, attenuated total reflection (ATR)-Fourier transform infrared (FT-IR) spectroscopy was conducted. The measurements were performed with a Nicolet iS5 Thermo Scientific spectrophotometer equipped with an ATR attachment equipped with a diamond crystal. The absorbance spectra were acquired over a range of 400–4000 cm^−1^ at ambient temperature.

### 3.7. SEM Analysis

The microstructure and surface morphology of the obtained polymer films were evaluated by a Tescan Mira 3 scanning electron microscopy instrument equipped with an FEG Schottky electron emission source at an acceleration voltage of 3.0 kV. The hydrogel specimens were sputter coated with a thin layer of gold for 30 s to improve surface conductivity.

### 3.8. Thermal Analysis

Thermogravimetric analysis was conducted using a Netzsch TG 209 F1 Libra apparatus. The measuring temperature ranged from 30 °C to 900 °C at a heating rate of 10 °C∙min^−1^ under a nitrogen atmosphere. The measurements were performed on samples with a mass of 10 ± 0.1 mg placed in Al_2_O_3_ crucibles. Moreover, differential scanning calorimetry (DSC) was applied to evaluate the thermal properties of the hydrogel materials. The measurement was performed using a Netzsch DSC 204 F1 Phoenix apparatus. Hydrogel samples with a mass of 10 ± 0.1 mg, placed in aluminum crucibles sealed with lids, were heated from −30 °C to 300 °C, at a rate of 10 °C∙min^−1^ in a nitrogen atmosphere.

### 3.9. Static Tensile Test

Static stretching tests were performed on the hydrogels using an MTS Bionix machine with a constant tensile loading rate of 0.2 mm/s. All specimens were prepared into a specific paddle shape (75 mm long, 4 mm at the middle, and 25 mm of measuring segment) using a blanking die. A film test was performed in a dry state. All tests were performed in accordance with the EN ISO 527:2 standard: Plastics—determination of tensile properties and results were recorded until the deformation limits were exceeded—i.e., to loss of sample integrity.

### 3.10. Cell Culture and Cytotoxicity Studies

Normal human dermal fibroblasts (NHDF) were bought from PromoCell. The NHDF cell line was cultured in Dulbecco’s Modified Eagle’s Medium (DMEM) supplemented with 15% non-inactivated fetal bovine serum (FBS) and contained a 1% *v/v* mixture of antibiotics: penicillin/streptomycin (Gibco). The cells were cultured under standard conditions: 37 °C in a humidified atmosphere with 5% CO_2_. Before the cytotoxicity experiments, the tested hydrogels were prepared as discs of approximately 2 cm diameter and were placed in PBS to remove excess solvent used in the synthesis. Then, the discs were transferred into a 12-well cell culture plate (Nunc) and dried for 24 h at room temperature. Finally, the hydrogel discs were sterilized with 70% ethanol and irradiated with a UV lamp. After preparation of the hydrogel materials, the fibroblast cells were seeded onto the discs at concentrations of 50,000 cells/well in 2 mL culture medium and incubated at 37 °C for 72 h. After this time, the metabolic activity of viable cells was determined by an MTS test. For this purpose, the culture medium was removed and replaced with 1 mL of DMEM without phenol red and 200 μL of CellTiter 96AQueousOne Solutions—MTS (Promega). After 1 h of incubation at 37 °C, the absorbance of the formed formazan in the samples was measured at 490 nm using a Synergy4 (BioTek) multi-plate reader. Additionally, a “blank” probe (MTS with DMEM) was detected. Each material was triplicate tested in a single experiment, while each experiment was repeated at least three times.

### 3.11. Cell Adhesion Assay

Before the bioimaging experiments, the tested materials were prepared as described above. Then, the fibroblast cells were seeded onto discs placed in a 35 mm imaging dish with a polymer coverslip at a concentration of 50,000 cells/well in 2 mL culture medium and incubated at 37 °C for 72 h. After this time, the culture medium was replaced with a 5 μM dye solution (CellTracker Green CMFDA) and incubated at 37 °C for 1 h. Afterwards, the hydrogel discs were washed three times with PBS. Visualization of stained cells on the hydrogel discs was carried out using a Zeiss Axio Observer.Z1 inverted fluorescence microscope equipped with an AxioCamMRm camera.

## 4. Conclusions

Generally, in the case of dermatological applications, such as transdermal systems, wound dressing, emulsions, or other cosmetics, the presence of glycerin is crucial because it increases the absorption of active substances through the skin and moisturizes it. Taking these facts into consideration, glycerin was introduced into the hydrogel matrix to produce a better carrier of active substances in the context of future applications as modern dressings. Based on the obtained results, we proved that the presence of glycerin in the structure of hydrogels directly influenced their properties. A higher content of glycerin significantly decreased in the gel fraction from 80.5 ± 2.1% to 45.0 ± 1.2%, which influenced the degree of material swelling—the materials enriched with polyol swelled less abruptly. Furthermore, they were more elastic and flexible, which could also be caused by changes in hydrogel structure during incomplete crosslinking due to the presence of glycerin in the matrix. The swelling ratios in tested fluids were very similar and reached about 200–300%. However, a trend of a decrease in swelling capacity after the addition of glycerin was noticeable. Degradation tests indicated that most of the tested materials were not degraded throughout the incubation period and maintained a constant ion level after 7-day incubation. The pH analysis of water and PBS fluid over time made it possible to draw a general conclusion that all analyzed materials enriched in glycerin led to a gradual pH decrease over time, while the absence of this component did not significantly affect the pH throughout the analyzed period. Generally, the presence of glycerin did not considerably influence the location of the peaks in the FT-IR spectra, however, the changes in intensity or presence of some of such peaks confirmed notable changes in the structure and alterations in the crosslinking process. Interestingly, the presence of glycerin in the polymeric matrices significantly decreased thermal resistance, which was especially visible in the case of T_10_ (from 273.9 °C to 163.5 °C). Moreover, all the tested hydrogel materials were characterized by a medium maximum deformation rate of around 37.6–69.5% at break. The proposed hydrogel materials containing sodium alginate (2% of solution), poly(vinyl alcohol) (5% of solution), and different amounts (0–3.4%, *v/v*) of glycerin showed no toxicity towards normal human dermal fibroblasts (NHDF) and did not induce a substantial decrease in their viability. This is the most important result because this composition of hydrogel matrix and type of preparation method can be used for further research, which involves multi-compartment dressing materials.

## Figures and Tables

**Table 1:** Effect of hydrogel composition on gel fraction (GF%) (*n* = 3).

Sample Symbol	S1G1	S2G1	S3G1	S4G1	S2G0.5	S2G0
GF [%]	45.0 ± 1.2	52.7 ± 1.9	55.9 ± 0.8	59.6 ± 0.8	63.4 ± 1.8	80.5 ± 2.1

**Table 2:** Thermal degradation profiles of hydrogels.

Sample Symbol	T_5_ [°C]	T_10_ [°C]	T_50_ [°C]	T_f_ [°C]	Residual Mass [%]
S1G1	88.7	153.7	153.7	412.8	4.4
S2G1	106.0	163.5	163.5	397.2	7.8
S3G1	111.3	172.5	172.5	415.7	4.4
S4G1	117.6	183.7	183.7	412.9	6.1
S2G0.5	109.1	171.1	171.1	408.5	6.6
S2G0	161.5	273.9	273.9	411.2	6.0

Temperatures at which 5%, 10%, and 50% weight loss was recorded by TG at heating rate 10 °C·min^−1^ in N_2_ atmosphere, respectively.

**Table 3:** Chemicals and other substances used in the experiments.

Substrate	Producer	Purity Degree
Sodium alginate	Sigma-Aldrich Inc.	Reagent grade
Poly(vinyl alcohol) (Mw 72,000 g/mol)	Avantor Performance Materials Poland S.A.	Reagent grade
Diacrylate poly(ethylene glycol) Mn. 700 (PEGDA)	Sigma-Aldrich Inc.	Reagent grade
Ammonium persulphate	Avantor Performance Materials Poland S.A.	Reagent grade
Glycerin	Avantor Performance Materials Poland S.A.	Reagent grade
Phosphate buffered saline pH 7.4 ± 0.2	OXOID™	n.d.

**Table 4:** Composition of SA/PVA hydrogels.

Sample Symbol	PVA Concentration [%]	SA Concentration [%]	Glycerin Content [%]
S1G1	5	1.5	3.4
S2G1	5	2	3.4
S3G1	10	1.5	3.4
S4G1	10	2	3.4
S2G0.5	5	2	1.7
S2G0	5	2	0.0

## Data Availability

The data that support the findings of this study are contained within the article.

## Supplementary Materials

The following are available online at https://www.mdpi.com/article/10.3390/ijms222112022/s1.
