# Supplementary figures and images for: The Effect of Glycerin Content in Sodium Alginate/Poly(vinyl alcohol)-Based Hydrogels for Wound Dressing Application
Source: Int J Mol Sci. 2021 Nov 6;22(21):12022. doi: 10.3390/ijms222112022 (PMC8584732; doi:10.3390/ijms222112022)

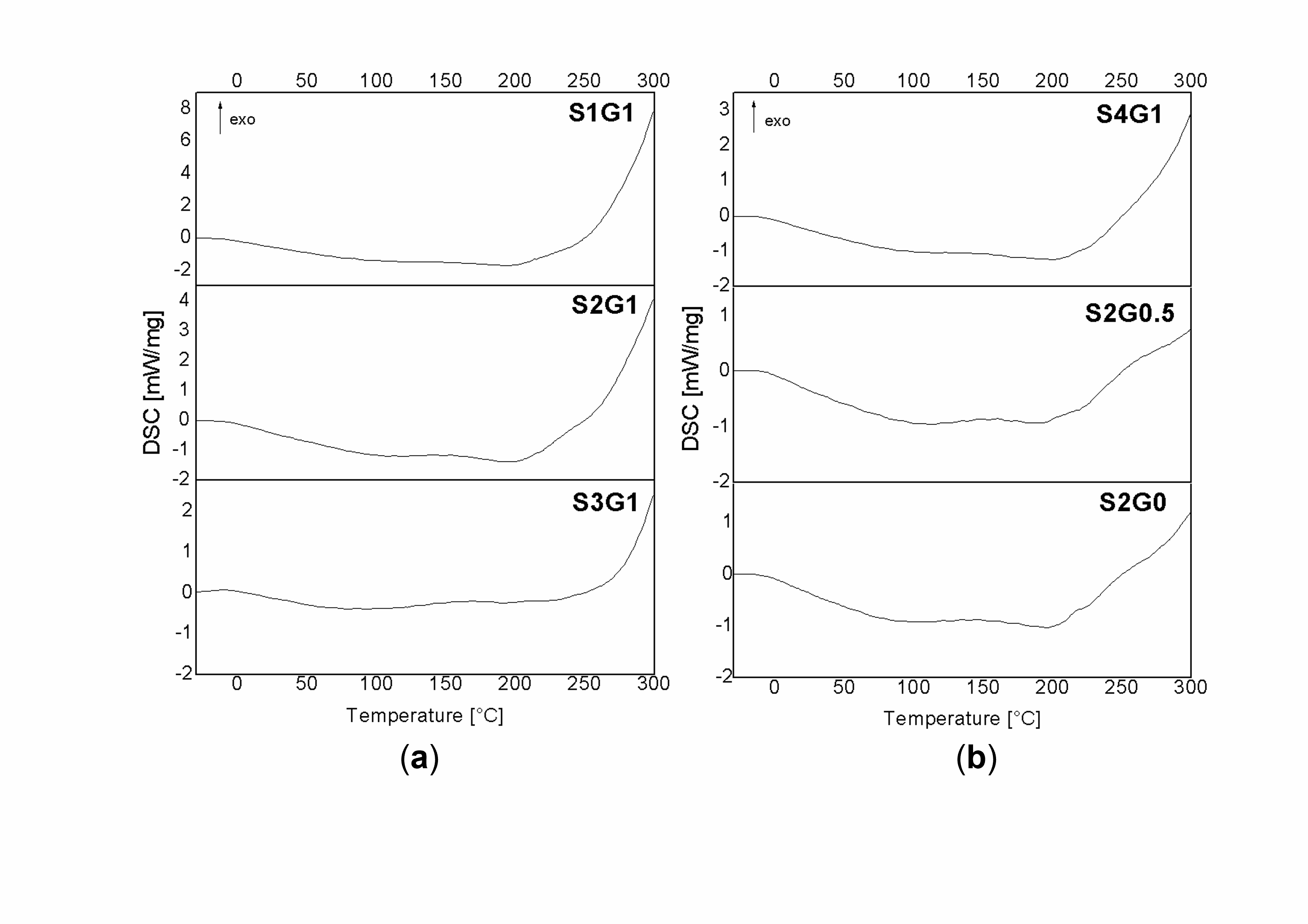

Supplement: Supplementary file 1 [file ijms-22-12022-s001.zip › ijms-1421010-SI.tif]
